# Avoiding Catch-22: validating the Pain*DETECT* in a in a population of patients with chronic pain

**DOI:** 10.1186/s12883-018-1094-4

**Published:** 2018-06-29

**Authors:** Hans Timmerman, André P. Wolff, Ewald M. Bronkhorst, Oliver H. G. Wilder-Smith, Marcel J. Schenkels, Nick T. van Dasselaar, Frank J. P. M. Huygen, Monique A. H. Steegers, Kris C. P. Vissers

**Affiliations:** 1 Department of Anesthesiology, Pain and Palliative Medicine, Radboud university medical center, Huispost 549, PO Box 9101, 6500 HB Nijmegen, the Netherlands; 2 Department of Anesthesiology, Pain Center, University of Groningen, University medical center Groningen, Groningen, the Netherlands; 3 Department for Health Evidence, Radboud university medical center, Nijmegen, the Netherlands; 4 Center for Sensory-Motor Interaction, Aalborg University, Aalborg, Denmark; 5 Bernhoven Ziekenhuis, Department of Anesthesiology, Uden, the Netherlands; 6 Reinier de Graaf Gasthuis, Department of Anesthesiology, Pain Medicine and Palliative Care, Delft, the Netherlands; 7 ErasmusMC, Department of Anesthesiology, University Center of Pain Medicine, Rotterdam, the Netherlands

**Keywords:** Pain*DETECT* questionnaire, Reliability, Validity, Sensitivity, Specificity, Screening tool, Neuropathic pain, Pain, Clinical assessment, Low back pain, Neck shoulder arm pain, Peripheral nerve damage

## Abstract

**Background:**

Neuropathic pain is defined as pain caused by a lesion or disease of the somatosensory nervous system and is a major therapeutic challenge. Several screening tools have been developed to help physicians detect patients with neuropathic pain. These have typically been validated in populations pre-stratified for neuropathic pain, leading to a so called “Catch-22 situation:” “a problematic situation for which the only solution is denied by a circumstance inherent in the problem or by a rule”. The validity of screening tools needs to be proven in patients with pain who were not pre-stratified on basis of the target outcome: neuropathic pain or non-neuropathic pain. This study aims to assess the validity of the Dutch Pain*DETECT* (Pain*DETECT*_*-Dlv*_*)* in a large population of patients with chronic pain.

**Methods:**

A cross-sectional multicentre design was used to assess Pain*DETECT*_-Dlv_ validity. Included where patients with low back pain radiating into the leg(s), patients with neck-shoulder-arm pain and patients with pain due to a suspected peripheral nerve damage. Patients’ pain was classified as having a neuropathic pain component (yes/no) by two experienced physicians (“gold standard”). Physician opinion based on the Grading System was a secondary comparison.

**Results:**

In total, 291 patients were included. Primary analysis was done on patients where both physicians agreed upon the pain classification (*n* = 228). Compared to the physician’s classification, Pain*DETECT*_-Dlv_ had a sensitivity of 80% and specificity of 55%, versus the Grading System it achieved 74 and 46%.

**Conclusion:**

Despite its internal consistency and test-retest reliability the Pain*DETECT*_-Dlv_ is not an effective screening tool for a neuropathic pain component in a population of patients with chronic pain because of its moderate sensitivity and low specificity. Moreover, the indiscriminate use of the Pain*DETECT*_*-Dlv*_ as a surrogate for clinical assessment should be avoided in daily clinical practice as well as in (clinical-) research. Catch-22 situations in the validation of screening tools can be prevented by not pre-stratifying the patients on basis of the target outcome before inclusion in a validation study for screening instruments.

**Trial registration:**

The protocol was registered prospectively in the Dutch National Trial Register: NTR 3030.

**Electronic supplementary material:**

The online version of this article (10.1186/s12883-018-1094-4) contains supplementary material, which is available to authorized users.

## Background

The International Association for the Study of Pain defines neuropathic pain as “pain caused by a lesion or disease of the somatosensory nervous system” and states that “neuropathic pain is not a medical diagnosis but a clinical description which requires a demonstrable lesion or a disease that satisfies established neurological diagnostic criteria” [1]. In the clinical context it is better to speak of a present or an absent neuropathic pain component (present- or absent- NePC) with respect to so called mixed-pain conditions [2, 3] in which neuropathic pain and nociceptive pain both exist. Clinically, NePC is considered to manifest specific symptoms and signs [4, 5]. The classification of NePC is usually based on history and physical examination including (bedside-) sensory testing [6, 7]. The correct classification of NePC is important for patients because NePC has a considerable impact on the quality of daily life [8] and for physicians since the treatment differs strongly from that of patients without NePC [6, 9, 10].

An easy to use and validated screening tool for clinical triage and epidemiological purposes could aid uniform classification and quantification of NePC and hence lead to better therapy, particularly when used by non-specialists [6–8, 11–15].

The Pain*DETECT* is such a patient friendly screening tool for the screening for neuropathic pain. It was originally developed and validated in Germany [2] based on two groups of patients (patients with pain of predominantly neuropathic origin or of predominantly nociceptive origin) with at least a 40% score on a visual analogue scale for pain (VAS;0–100). The gold standard used in this study was the assessment of the pain type based on the examination by two experienced pain specialists. This resulted in a percentage of correctly identified patients of 83% for neuropathic pain, a sensitivity of 85 and 80% specificity [2]. Subsequently, validation studies were performed in Spain [16], Turkey [17], Japan [18], India (Hindi) [19] and Korea [20]. Since the introduction of the Pain*DETECT* this instrument has been used in many clinical and epidemiological studies [21]. In a Danish study, based on Pain*DETECT* outcome, NePC was present [22] in about 40% of the patients with musculoskeletal pain.

In the above-mentioned validation studies [2, 16–20], the validity of the Pain*DETECT* as a screening tool was performed in *pre-stratified* groups of patients based on the target outcome (pain of predominantly neuropathic origin or of predominantly nociceptive origin and limitation to pain scores). The inclusion of only patients with a known pain classification on forehand might lead to a prerequisite for the determination of validity of the PainDETECT. For this situation, the term “Catch-22” is used in the English language for“* a problematic situation for which the only solution is denied by a circumstance inherent in the problem or by a rule”* [23]. It was firstly described in Joseph Heller’s novel Catch-22 which describes a general situation in which an individual has to accomplish two actions that are mutually dependent on the other action that must be completed first.

The objective of this study is to further validate the Pain*DETECT* as a screening tool for use in daily outpatient practices for detecting a NePC. The current validation study is being conducted in a general patient population having common chronic pain syndromes, not pre-stratified on the target outcome: low back with leg pain (LBLP), neck-shoulder-arm pain (NSA pain) or a suspected peripheral nerve damage pain (suspected PND pain).

## Methods

The study was conducted in a cross-sectional, observational, research design with two weeks and three months follow up to study the clinimetric quality (i.e. reliability and validity) of the Pain*DETECT*. This study, to detect a NePC in patients suffering from chronic pain, was approved by the medical and ethical review board Committee on Research Involving Human Subjects region Arnhem-Nijmegen, Nijmegen, the Netherlands, Dossier number: 2008/348; NL 25343.091.08 and conducted in accordance with the declaration of Helsinki and the declaration of the World Medical Association. As required, written informed consent was obtained from patients prior to study participation. The protocol is registered in the Dutch National Trial Register: NTR 3030. The Pain*DETECT* was translated and cross-culturally adapted into the Dutch language (Pain*DETECT*_-Dlv_) (© Pfizer Pharma GmbH 2005, Pfizer bv 2008. Cappelle a/d IJssel, the Netherlands) in a separate study [24] before the commencement of the present validation study. In this study, the same methodology was used as in the previously published protocol [25] and as employed in a simultaneous study regarding the validity of the DN4 [26] .

### Patients

The patients were recruited from October 2009 until July 2013. Multicenter recruitment took place in the Netherlands in three academic centers specialized in pain medicine, three non-academic centers specialized in pain medicine and one non-academic department of neurology. The question to participate in the study was asked by the patients’ own physician. At that moment they only had a provisional diagnosis: LBLP, NSA pain or pain due to a suspected PND (Conditions associated with a lesion of the peripheral somatosensory system). These three groups of patients include a majority of the patients referred towards an academic or peripheral pain clinic from the general practitioner. Patients had to be diagnosed for the initial cause of the pain as classified according to the International Statistical Classification of Diseases and Related Health Problems 10th Revision (ICD-10)-2015-WHO Version 2015 [27]. Importantly, patients were not pre-stratified on the target outcome: the existence of NePC yes or no [28]. Patients, when willing to participate, were included when they met the following inclusion criteria: Male or female adult patients (> 18 years of age) with chronic (≥3 months) LBLP or NSA pain radiating into leg (s) or arm (s) respectively or patients with chronic pain due to a suspected PND. Exclusion criteria were*:* Patients diagnosed with an active malignant disorder, compression fractures, patients with diffuse pains (pains with an origin in muscles, bones or joints: such as fibromyalgia or ankylosing spondylitis), severe mental illness, chronic alcoholism or substance abuse, inability to fill in the questionnaire adequately or incapable of understanding the Dutch language.

### Physicians’ assessment

Patients were examined for the presence of NePC by two physicians which was considered to be the “gold standard” in this study. The physicians (pain specialists, pain specialist in training or neurologists always operated in differently composed pairs) worked independently from each other and were blinded to the classification made by the other physician. The physicians were not selected on basis of age, years of experience as a physician or other criteria. A full medical history was taken followed by a thorough clinical examination. A bedside examination (touch, pin prick, pressure, cold, heat and temporal summation) to assess patients’ pain [25] was based on the European Federation of Neurological Societies (EFNS) guidelines [29, 30], the IASP Neuropathic Pain Special Interest Group (NeuPSIG) guidelines on neuropathic pain assessment [6] and the guidelines for assessment of neuropathic pain in primary care [7]. Patients’ pain was classified by the physician as pain with present- or absent-NePC. The NeuPSIG Grading System for neuropathic pain as proposed by Treede et al. [31] was used as a secondary comparison with the outcome of the Pain*DETECT*_-Dlv_. The assessment of the Grading System was implemented in the standardized assessment protocol and thus included in the diagnostic work-up of the patients [25]. The outcomes “probable” and “definite” were regarded as “present-NePC”. “Unlikely” and “possible” were rated as “absent-NePC” [32–34]. All participating physicians underwent standard medical training, belonging to the classic medical curriculum, and examination of the (central) nervous system in particular. To achieve standardization of history and assessment of NePC presence in patients included in this study all participating physicians underwent a training in the performance of the clinical examination of the patients (including sensory (bedside) examination and use of the NeupSIG Grading System) [25]. Training of the physicians took place at the participating center. During the execution of the study, the study coordinator (HT) visited the participating centers on a regularly basis to answer questions, to see if the necessary equipment was always available and to keep an eye on the inclusion of patients. Based on the order of assessment, the physician who performed the first assessment was called physician A and the physician who performed the assessment as a second physician was named physician B. However, the order of the physicians was based on availability during the study.

#### PainDETECT_-Dlv_ and other questionnaires

The Pain*DETECT*_-Dlv_ (© Pfizer Pharma GmbH 2005, Pfizer bv 2008. Cappelle a/d IJssel, the Netherlands) [2, 24] was designed as a simple, patient self-administered screening tool to screen for the presence of neuropathic pain without physical examination. This instrument consists of one item about the pain course pattern, one about radiating pain and seven items about the gradation of pain. An overall score is generated and ranges between − 1 and 38. Additionally, there are three items about pain severity (current, worst and average pain) included in the Pain*DETECT*. For the original German version [2] the outcome was as follows: ‘-1 - 12: negative’, neuropathic pain is unlikely; 13–18: ‘unclear’; result is ambiguous, however neuropathic pain can be present; 19–38 ‘positive’, neuropathic pain is likely.

The patient completed five questionnaires (including the Pain*DETECT*_-Dlv_ directly after the clinical assessment by the participating physicians but without any interference by the physicians. The researcher (HT) was available for help via telephone or in person when it was not clear how to fill in the questionnaires. Besides screening for NePC via the Pain*DETECT*_-Dlv_ [24], the disability of the patient was assessed via the Disability Rating Index (DRI) [35]. The existence of an anxiety disorder and/or depression were assessed via the Hospital Anxiety Depression Scale (HADS) [36–38] and the Pain Attribution Scale (PAS) was used to assess patients attribution of his or hers pain. Quality of life was determined via the RAND 36-item Health Survey (RAND-36) [39–41]. Two weeks and three months after the initial visit the follow-up questionnaires (the Patients Global Impression of Change (PGIC) [42–44] and the Pain*DETECT*_-Dlv_) were sent to the patient by mail.

### Data

All data gathered from patients and physicians was collected on paper and stored at the Radboudumc, Nijmegen, The Netherlands. Data management and monitoring were performed within MACRO (MACRO, version 4.1.1.3720, Infermed, London, United Kingdom).

### Statistical methods

Power calculation for this study was based on a expected NePC prevalence of 37% in an unselected cohort of patients with chronic low back pain [2]. Sensitivity and specificity of the Pain*DETECT* were assessed in the original validation study as respectively 85 and 80% [2]. The sensitivity and specificity of the Pain*DETECT*_-Dlv_ was, on forehand, expected to be 80% with a prevalence of 37%. The lower 95% confidence limit was required to be > 0.55. According to the calculations following the formulas by Flahault et al. [45] 132 patients with LBLP, NSA pain or suspected PND pain were needed so that the sample size contained a sufficient numbers of cases and controls [25].

Qualitative variables were presented as frequencies and percentages. The quantitative variables were presented as mean and standard deviation (SD) or as median and inter quartile range (IQR). Based on the classifications of the two physicians, all patients were categorized as absent-NePC, NePC or ‘undetermined’ (i.e. the classification by both physicians jointly was not equal).

One-way ANOVA (with additional Tukey’s studentized range post-hoc test) or Kruskal-Wallis test were used to study differences between the three groups (NePC, absent-NePC, Undetermined).

Intraclass correlation (ICC) was used to assess reproducibility (‘test-retest reliability’) of the Pain*DETECT*_-Dlv_ between the fixed time points (baseline versus two weeks & baseline versus three months)_._ The ICC and responsiveness of the Pain*DETECT*_-Dlv_ were assessed between each point of measurement.

A receiver operating characteristic (ROC) curve was calculated and the area under the curve (AUC) with 95% confidence interval is presented to indicate the discriminatory power of the Pain*DETECT*_-Dlv_ to discriminate patients classified as with or without a NePC. The classification was based on the physicians’ assessment outcome or based on the Grading System outcome, respectively. The theoretical maximum of the AUC is 100%, indicating a perfect discrimination and 50% is equal to tossing a coin. The optimal cut-off point of the Pain*DETECT*_-Dlv_ – sum score was calculated under the condition of equal-costs of misclassification, using the Youden-index. Sensitivity, specificity, positive and negative predictive values and the likelihood ratio in the population in this study was calculated at this cut-off point. Also, the ‘number needed to diagnose (NND)’ was assessed [46] by use of the formula: NND = 1/[Sensitivity – (1-specificity)]. A clinical screening tool for the demonstration of a neuropathic pain component is considered valid if it has a high sensitivity, specificity and a high positive predictive value. For the measurement of the usefulness of the screening tool the likelihood ratio will be used [47].

The agreement between the pain classification by the physicians, the NeuPSIG Grading Systems and the Pain*DETECT*_-Dlv_ (yes: ≥11, no:< 11) outcome was evaluated by using Cohen’s kappa (*K*), prevalence index (P*i*) and percentage of pair wise agreement (PA) [25]. A *Κ* ≥ 0.40 and a PA ≥ 70% is considered indicative of interobserver reliability which is acceptable for use in clinical practice [48].

Data analysis and statistics were performed by use of Statistical Package for the Social Sciences (SPSS version 20.0, SPSS Inc., Chicago, Illinois, USA). Two-tailed *p*-value below 0.05 was considered statistically significant.

## Results

### Patient population

In this study 330 patients, not pre-stratified on the target outcome, with chronic LBLP, NSA pain or suspected PND pain were assessed for eligibility. Two patients did not give their informed consent. Exclusion (*n* = 37) was due to not fulfilling the in- and exclusion criteria (*n* = 13); not returning the baseline questionnaires by the patient (*n* = 16); missing pain classification by one physician (*n* = 5) or both physicians (n = 3). In eight patients the assessment of the grading system (secondary comparison) was missing by one or both physicians. Finally, 291 patients participated in the study between October 2009 and July 2013. According to the international classification of diseases (ICD-10, version 2015) [27] these patients were classified as follows: 8 patients suffered from pain related to endocrine, nutritional and metabolic diseases (chapter IV); 75 patients from diseases of the nervous system (chapter VI); 1 patient from diseases of the circulatory system (chapter IX);189 patients from diseases of the musculoskeletal system and connective tissue (chapter XIII); 1 patient from diseases of the genito-urinary system (chapter XIV); 3 patients from symptoms, signs and ill-defined conditions, and 14 patient from injury, poisoning or other consequences of external causes.

**Fig. 1:**
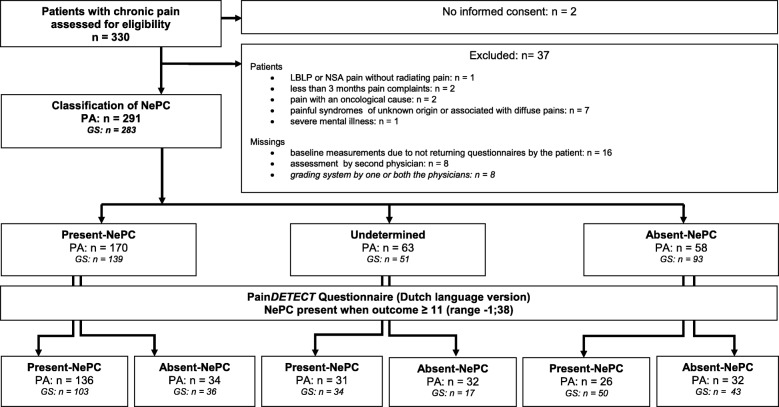
Flow diagram for the validation of the PainDETECT_-Dlv_ . PA: Physicians’ assessment; GS: Neuropathic pain special interest group Grading System; Present-NePC: Neuropathic pain component present; Undetermined: both physicians disagree with each other about the presence of a neuropathic pain component; Absent-NePC: No neuropathic pain component present; n = number of patients in analysis

Numbers of recruitment in the different participating hospitals (all in the Netherlands) were as follows: Reinier de Graaf Gasthuis, Delft *n* = 86; ErasmusMC, Rotterdam *n* = 62; Radboudumc, Nijmegen *n* = 59; Bernhoven Ziekenhuis, Oss *n* = 56; Rijnstate Ziekenhuis, Arnhem *n* = 15; St. Anna ziekenhuis, Geldrop *n* = 12 and UMC Utrecht, Utrecht *n* = 1. 132 patients had LBLP with radiation in one or two legs (45.4%), 51 NSA pain with radiation into one or both arms (17.5%) and 108 (37.1%) had suspected PND pain. The group of patients with suspected PND consisted of 86 patients with pain who were treated because of breast cancer (surgery and/or radiation and/or chemotherapy and/or hormonal therapy). The remaining 22 patients had pain because of various reasons: peripheral nerve damage (*n* = 12), polyneuropathy (*n* = 3), central post stroke pain (*n* = 2), Complex Regional Pain Syndrom (n = 2) and spinal radicular pain (*n* = 3). After assessment by physicians A and physicians B, 170 patients were classified as having present-NePC, 58 as absent-NePC. In 63 patients the two physicians made a non-concordant pain classification, so the outcome based on the physicians assessment was classified as ‘undetermined’. Based on the NeuPSIG Grading System in 139 patients NePC was classified as present, in 93 patients NePC was absent and in 51 patients the two physicians made a non-concordant pain classification in which the outcome was classified as ‘undetermined’ (see Fig. 1: Flow Diagram).

Social-demographic and clinical details of the 291 patients were analyzed and divided from each other based on the pain classification (see Table 1). No statistically significant differences were found between absent-NePC, present-NePC and undetermined for gender, age, height, weight, body mass index (BMI), education, medication, duration of pain, quality of life, disability, pain attribution, anxiety disorder and depression. Moreover, no statistically significant differences were observed between absent-NePC and present-NePC for pain (current, worst and average pain).

**Table 1:** Socio-demographic and clinical characteristics of the patients related to physicians agreement for the existence of a NePC

NePC	Absent		Present		Undetermined	
	N	n (%)	N	n (%)	N	n (%)
		Mean (±SD)		Mean (±SD)		Mean (±SD)
		Median [IQR]		Median [IQR]		Median [IQR]
Gender	58		170		63	
Male		25 (43%)		56 (33%)		17 (27%)
Female		33 (57%)		114 (67%)		46 (73%)
Age (Years)	58	55 ± 12	170	56 ± 11	63	58 ± 13
Height (cm)	55	172 ± 9	164	172 ± 8	62	170 ± 9
Weight (kg)	55	84 ± 25	167	80 ± 17	62	80 ± 16
BMI (kg/m^2^)	54	28 ± 8	164	27 ± 5	62	27 ± 5
Education	56		164		63	
Functional illiterate		0 (0%)		0 (0%)		2 (3.3%)
Primary education		2 (3.6%)		14 (8.5%)		6 (9.8%)
Secondary education		32 (57.1%)		98 (59.8%)		38 (62.3%)
Postgraduate		22 (39.3%)		52 (31.7%)		15 (24.6%)
Medication (% yes)	55	31 (56.9%)	168	111 (66.1%)	61	35 (57.4%)
Pain (NRS; 0–10)						
Current pain	57	5 [3–7]	167	6 [3–7]	61	4 [1–7]
Worst pain (past four weeks)	57	8 [5–9]	167	8 [7–9]	61	7 [5–8]
Average pain (past four weeks)	57	6 [3.5–7]	167	6 [5–8]	61	6 [3–7]
Duration of pain (months)	57	72 ± 90	169	60 ± 76	62	49 ± 46
Quality of life						
Physical functioning	58	57 ± 27	170	51 ± 25	62	55 ± 29
Role functioning physical	58	43 ± 42	170	35 ± 41	61	41 ± 45
Role functioning emotional	58	80 ± 35	169	70 ± 43	61	73 ± 42
Social functioning	58	43 ± 14	170	44 ± 11	62	46 ± 10
Bodily pain	58	55 ± 24	170	56 ± 25	62	46 ± 25
Mental health	58	60 ± 6	170	61 ± 10	61	62 ± 7
Vitality	58	51 ± 10	170	49 ± 12	61	50 ± 11
General health	57	58 ± 14	165	57 ± 14	60	55 ± 12
Health change	58	38 ± 24	170	40 ± 26	63	42 ± 27
Disability						
Total	53	46 ± 27	158	48 ± 24	57	40 ± 26
Pain attribution						
Somatic	53	5.2 ± 4.3	156	6.0 ± 4.0	58	5.2 ± 3.9
Psychological	58	2.0 ± 2.9	164	2.2 ± 3.2	60	2.9 ± 3.0
Social	57	1.6 ± 2.2	163	2.0 ± 2.6	61	2.4 ± 2.6
Anxiety disorder	57	14 (24.6%)	167	46 (27.5%)	60	18 (30.0%)
Depression	57	14 (24.6%)	166	46 (27.7%)	61	11 (18.0%)

Classification for the existence of NePC is based on physicians assessment of the patients

*NePC: neuropathic pain component; Absent: NePC is absent; Present: NePC is present; Undetermined: both physicians disagree with each other about the existence of a neuropathic pain component*; *N: total number of patients in analysis; n: number of patients; %: percentage; SD: Standard deviation; IQR: Inter quartile range*

### Physicians

During this study 62 various physicians (pain specialist, pain specialist-fellow or neurologist), from seven different hospitals, assessed all included patients. All patients were assessed two times by two different physicians. Of all participating physicians, 21 physicians assessed ≤2 patients during the execution of the study, 23 physicians saw ≤9 patients, 10 physicians saw ≥10 patients and 8 physicians saw ≥20 patients.

### Evaluation of the PainDETECT_-Dlv_

The mean score of the Pain*DETECT*_-Dlv_ (Range − 1;38) for patients classified as absent-NePC was 10.7 (SD ± 5.7); for patients classified as present-NePC it was 15.7 (SD ± 6.3) and for patients with an undetermined outcome it was 11.8 (SD ± 5). As calculated based on a one-way ANOVA with Tukey’s studentized range post-hoc test, there was a statistical significant difference between absent-NePC and present-NePC (*P* < 0.001) and between present-NePC and undetermined (*P* < 0.001). No significant difference was seen between absent-NePC and undetermined (*P* = 0.57). Patients pain course pattern and if the pain was radiating to other regions of the body were not significantly different between the three groups. Pain descriptors (burning, tingling or prickling, painful light touching, sudden pain attacks, temperature evoked pain, numbness sensation and pressure evoked pain) were all statistically significant discriminators for the presence of NePC (*P* ≤ 0.005) except for pressure evoked pain (*P* = 0.07). See Table 2 for the Pain*DETECT*_-Dlv_ outcomes divided according to the pain classification by the physicians (present- NePC, absent-NePC or undetermined) (See Table 2).

**Table 2:** The median (IQR) of the items of the Pain*DETECT* by physicians agreement for the existence of a NePC

	NePC	Absent		Present		Undetermined	
PainDETECT item		N	n (%)	N	n (%)	N	n (%)
			Median [IQR]		Median [IQR]		Median [IQR]
			Mean (±SD)		Mean (±SD)		Mean (±SD)
Pain course pattern		58		162		59	
Persistent pain with slight fluctuations			19 (33%)		53 (33%)		17 (29%)
Persistent pain with pain attacks			14 (24%)		58 (36%)		17 (29%)
Pain attacks without pain between them			16 (28%)		32 (20%)		20 (34%)
Pain attacks with pain between them			9 (16%)		18 (11%)		5 (9%)
Radiating pain (% yes)		51	41 (78%)	154	112 (73%)	57	38 (67%)
Gradation of pain							
Burning		55	0 [0–2]	170	1 [0–3]	62	0 [0–2.25]
Tingling or prickling		55	1 [0–3]	170	2 [0–3]	63	1 [0–3]
Painful light touching		55	0 [0–1]	169	1 [0–2]	63	0 [0–1]
Sudden pain attacks		55	2 [0–3]	167	3 [1–4]	62	2 [0–3]
Temperature evoked pain		54	0 [0–1]	170	1 [0–2]	63	1 [0–1]
Numbness sensation		56	2 [0–3]	170	3 [2–4]	63	3 [1–4]
Pressure evoked pain		55	2 [1–3]	170	3 [1–4]	63	2 [1–3]
Total sum score Pain*DETECT*		58	10 [6.75–15.25]	170	16 [11–20]	63	10 [8–15]
			10.7 (± 5.75)		15.7 (± 6.3)		11.8 (± 5)

Classification of NePC is based on physicians assessment of the patients

*NePC: neuropathic pain component; Absent: NePC is absent; Present: NePC is present; Undetermined: both physicians disagree with each other about the existence of a neuropathic pain component*; *N = total number of patients in analysis; n = number of patients; IQR: inter quartile range; SD: standard deviation; Range: 0 = never; 1 = hardly noticed; 2 = slightly; 3 = moderately; 4 = strongly; 5 = very strongly; Total sum score PainDETECT: Sum score calculation of the PainDETECT*

### Validity

**Fig. 2:**
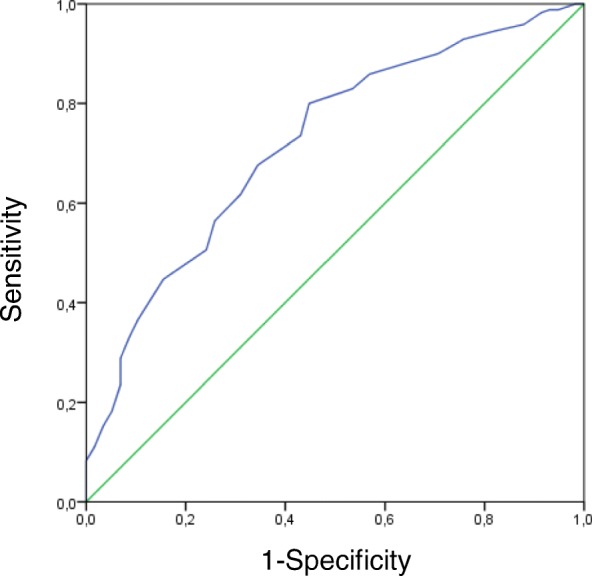
Receiver operating characteristics and area under the curve (AUC) for the total score of the PainDETECT_-Dlv_ versus the presence of a neuropathic pain component as classified by two physicians (*n* = 228; undetermined patients are not included). X-axis: 1-Specifity; Y-axis: Sensitivity

The gold standard for presence of the NePC in this study was the concordant opinion of both physicians. On basis of this gold standard, patients with an identical pain classification were included in the initial analysis (*n* = 228): 58 patients were classified as absent-NePC (25.4%) and 170 were classified as present-NePC (74.6%)(see Table 3 and Additional file 1: Table S1). A ROC-curve was constructed for Pain*DETECT*_-Dlv_ (see Fig. 2). Based on the gold standard, Pain*DETECT*_-Dlv_ sensitivity was (at maximal Youden-index) 80%, specificity 55.2%, positive predictive value 84% and the positive likelihood ratio was 1.78. Based on the neuropathic pain Grading System, the sensitivity was 74.1%, specificity 46.2%, positive predictive value 67.3%, and positive likelihood ratio of 1.38.

**Table 3:** The AUC and the sensitivity / specificity at the optimal cut-off point of the PainDETECT under the condition of equal costs of misclassification to classify NePC by the classification and the Grading System of the physicians

Pain*DETECT* versus classification by:	NePC:		AUC %	(95%CI)	Cut -off	Sens %	(95% CI)	Spec %	(95% CI)
	Absent (n)	Present (n)							
Assessment A	83	208	69.8	(0.63–0.77)	9	86.0	(0.81–0.90)	45.8	(0.36–0.57)
Assessment B	96	195	67.2	(0.61–0.74)	11	75.4	(0.69–0.81)	52.1	(0.42–0.62)
Assessment A = Assessment B	58	170	72.1	(0.65–0.80)	11	80.0	(0.73–0.85)	55.2	(0.43–0.67)
*LBLP*	28	75	75.4	(0.65–0.86)	11	84.0	(0.74–0.91)	64.3	(0.46–0.79)
*NSA pain*	18	23	62.9	(0.46–0.80)	9	82.6	(0.63–0.93)	44.4	(0.25–0.66)
*Suspected PND pain*	12	72	75.5	(0.63–0.88)	15	55.6	(0.44–0.67)	91.7	(0.65–0.99)
Grading A	114	172	58.9	(0.52–0.66)	11	73.3	(0.66–0.79)	43.9	(0.35–0.53)
Grading B	130	158	58.6	(0.52–0.65)	14	57.6	(0.50–0.65)	59.2	(0.51–0.67)
Grading A = Grading B	93	139	61.3	(0.54–0.69)	11	74.1	(0.66–0.81)	46.2	(0.37–0.56)
*LBLP*	60	48	63.1	(0.52–0.74)	12	70.8	(0.57–0.82)	55.0	(0.43–0.67)
*NSA pain*	24	13	48.9	(0.28–0.69)	18	30.8	(0.13–0.58)	87.5	(0.69–0.96)
*Suspected PND pain*	9	78	66.0	(0.45–0.87)	13	64.1	(0.53–0.74)	77.8	(0.45–0.94)
Assessment A = Grading A	63	155	69.1	(0.61–0.77)	11	76.1	(0.69–0.82)	55.6	(0.43–0.67)
Assessment B = Grading B	77	139	67.1	(0.60–0.74)	11	76.3	(0.69–0.83)	52.0	(0.41–0.63)
Assessment A = Grading A = Assessment B = Grading B	43	118	67.9	(0.59–0.77)	11	78.0	(0.70–0.85)	53.0	(0.39–0.68)

Classification of NePC is based on (both) physicians’ assessment of the patients and / or on (both) the NeuPSIG Grading Systems

*NEPC: Neuropathic pain component existing; Absent NePC: Neuropathic pain component not existing; AUC: Area under curve; 95%CI: 95% confidence interval; Cut-off: Cut-off value; Sens.: Sensitivity; Spec.: Specificity; LBLP: Patients with low back and leg pain; NSA pain: Patients with neck-shoulder-arm pain; suspected PND pain: Patients with pain due to a suspected peripheral nerve damage*

We also constructed ROC-curves for the classification by solely physicians A or B and according to the neuropathic pain Grading System by physicians A or B and all the combinations. Except for classification of patients’ pain based on the description of physicians A and the outcome of the Grading System by physicians B all cut-off scores were calculated at 11-points out of 38: The sensitivity ranges from 57.6–86.1% and specificity from 43.9–59.2%. The classification of patients’ pain based on the classification of physicians A resulted in a cut-off score of 9-points: Sensitivity 86.1% and specificity 45.8%. The classification of patients’ pain based on the Grading System according to physicians B resulted in a cut-off score of 14-points: Sensitivity of 57.6% and specificity of 59.2%. In Table 3 and Additional file 1: Table S1 we present the number of patients with LBLP, NSA pain or suspected PND pain, in total and per group based on physicians’ assessment and/or the Grading System. Values of the AUC, cut-off value, sensitivity and specificity are provided (see also Additional file 1: Table S1 for a more detailed analysis of the diagnostic accuracy of the Pain*DETECT*_-Dlv_: AUC, cut-off value, sensitivity, specificity positive and negative predictive values, positive likelihood ratios and the number needed to diagnose (NND).

**Fig. 3:**
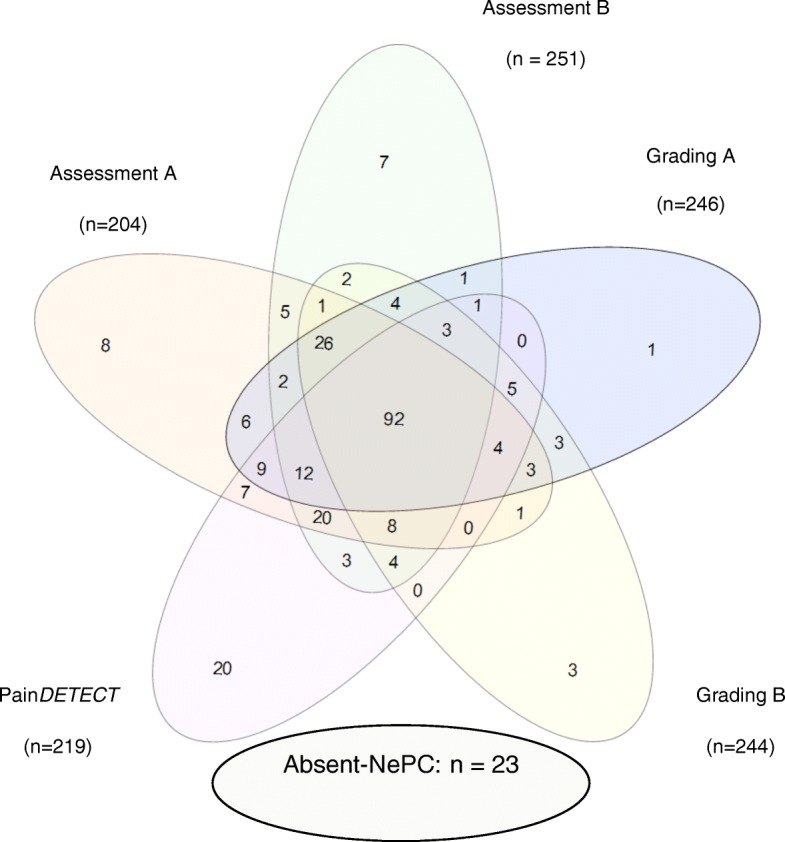
VENN-Diagram of all the five outcomes per patient. Physicians A: classification of a neuropathic pain component (NePC) exists; Physicians B: NePC exists; Grading A: NePC exists according to the Grading System by physicians A; Grading B: NePC exists according to the Grading System by physicians B; PainDETECT_-Dlv_: Outcome of the PainDETECT_-Dlv_ indicates the existence of a NePC. Non NePC: No NePC exists according to physicians, Grading Systems and the PainDETECT_-Dlv_

Patients were screened on a NePC (positive outcome) by two physicians, two times the Grading System, and the patient completed the Pain*DETECT*_-Dlv_. All the possible outcome combinations were computed based on the outcome: Is a NePC present, or not? In 283 patients all the five outcome variables were available and are displayed in a Venn-diagram [49] (see Fig. 3). In 92 patients (32.5%), five times a positive outcome variable was found, indicating presence of NePC. In 23 patients all outcome variables were negative (8.1%), thus indicating absence of NePC. One positive outcome was detected in 39 patients (13.8%), two positive outcomes in 28 patients (9.9%), three in 49 patients (17.3%), and four in 52 patients (18.4%).

### Reliability

To determine the interobserver reliability between the physicians, the Grading System and the outcome of the Pain*DETECT*_-Dlv_ for the classification of a (absent-) NePC, Cohen’s kappa (*K*) and percentage of pair wise agreement (PA) were assessed (see Table 4) *K* for the classification of patients’ pain (absent-NePC or NePC) by the physicians was 0.49, with a PA of 78.4% (P*i* = 0.38; *n* = 291). The *K* for the classification of patients’ pain based on the Grading System was 0.63 and PA was 82% (P*i* = 0.16; *n* = 283). The outcome of *K* and PA regarding the Pain*DETECT*_-Dlv_ compared to the classification of physicians A was respectively 0.34 and 74.6% (P*i* = 0.48; n = 291). Compared to physicians B it was 0.27 and 67.7% (P*i* = 0.33; n = 291). Comparing the outcome of the PainDETECT_-Dlv_ to the outcome of the Grading System, was 0.18 and 61.5%(P*i* = 0.27; *n* = 286) for physicians A, and 0.17 and 58.3%(P*i* = 0.05; *n* = 288) for physicians B.

**Table 4:** The kappa coefficient between the classification on basis of the assessment by the physicians, the Grading Systems and the Pain*DETECT*

		Pain*DETECT* (yes / no)	Grading A	Grading B	Assessment B
Assessment A	n	291	286	288	291
	*K*	0.34	0.48	0.32	0.49
	PA	74.6	76.2	67.4	78.4
	P*i*	0.48	0.32	0.26	0.38
Assessment B	n	291	286	288	
	*K*	0.27	0.38	0.48	
	PA	67.7	71.0	75.0	
	P*i*	0.33	0.28	0.22	
Grading A	n	286		283	286
	*K*	0.18		0.63	0.38
	PA	61.5		82.0	71.0
	P*i*	0.27		0.16	0.28
Grading B	n	288			
	*K*	0.17			
	PA	58.3			
	P*i*	0.05			

Classification of NePC is based on physicians’ assessment of the patients and on the Grading Systems

n = number of patients in the analysis; K = Cohen’s kappa coefficient; PA (%) = percentage of agreement between two outcome variables; Pi = Prevalence index

Stability and responsiveness of the PainDETECT_-Dlv_ over time was assessed over a period of two weeks. The mean sum score of the PainDETECT_-Dlv_ at baseline for the total group was 13.8 ± 6.3. The mean sum score after two weeks was 14.1 ± 6.1. Test-retest reliability via ICC was 0.83 (95%CI 0.79–0.87; *n* = 268). Taking into consideration the fact that patients’ pain should not have changed (outcome based on the PGIC), because otherwise the ICC would not reflect the consistency of the PainDETECT_-Dlv_, and a time gap of 7–21 days was allowed (to rule out the early or delayed return of questionnaires) between the first and second Pain*DETECT*_-Dlv_*,* the ICC was 0.87 (95% CI 0.81–0.91; *n* = 123). After three months, with no change in the degree of patients’ pain and a time gap of 60–120 days between the first and third Pain*DETECT*_-Dlv_, ICC was 0.86 (95% CI 0.79–0.91; *n* = 102).

## Discussion

This study demonstrates the clinimetric quality of the Pain*DETECT*_-Dlv_, a screening instrument for the presence of a NePC, on a large population of patients, with chronic pain due to low back with leg pain, neck-shoulder-arm pain or pain due to a suspected peripheral nerve damage as normally present in a physician’s daily practice. Because the patients were included without pre-stratification on the target outcome, previous Catch-22 situations in the assessment of the validity of screening instruments were avoided. Under these conditions, the Pain*DETECT*_-Dlv_ failed to be predictive for the existence of a NePC due to a moderate sensitivity and low specificity, irrespective of comparison with the expert opinion via the classification by two physicians (gold standard) as well as with the outcome of the NeuPSIG Grading System. Moreover, the predictive values were also not indicating that the Pain*DETECT*-_Dlv_ is a valid screening tool for the assessment of a NePC. The likelihood ratios were also not suggestive for the usefulness of this instrument.

### Validation studies with patients pre-stratified for NePC

We found an optimal cut-off score for the Pain*DETECT*_-Dlv_ of ≥11 points corresponding to a sensitivity of 80% and a specificity of 55%. In the original development and validation study of the Pain*DETECT* by Freynhagen et al. [2] a sensitivity and specificity of 84% was found. The gold standard in their study was the examination by two experienced pain specialists. The study was performed at ten different specialized pain centers. Only patients with ‘typical’ neuropathic or nociceptive entities (i.e. *no ‘unclear’ outcome*) and only patients with a VAS of > 40 mm (0 - 100 mm) were included. In the Spanish validation study by De Andrés et al. [16] only patients with a VAS ≥40 mm and a known classification (by one experienced specialist) of neuropathic pain, mixed pain or nociceptive pain were included. It revealed a sensitivity and specificity of 81% when patients with the classification of neuropathic pain or nociceptive pain were included. The inclusion of patients with mixed pain in the neuropathic pain group resulted in a sensitivity of 84% and specificity of 78%. The Korean version of the Pain*DETECT* [20] was validated based on the study by De Andrés [16] in patients with chronic pain and with a NRS ≥ 3 (NRS 0–10). The gold standard was the independent diagnosis of the patient by two experienced pain physicians. It revealed a sensitivity of 82% and a specificity of 92% based on a cut-off score of ≥19 (range − 1; 38). In the validation of the Turkish version of the Pain*DETECT* [17] patients were included with the classification of pain type (i.e. NePC) being assessed beforehand (based on the opinion of two expert pain physicians) and patients suffering from pain of three centimeters or more (VAS 0–10 cm). Sensitivity and specificity were respectively 78 and 83%. The Hindi version of the Pain*DETECT* [19] was validated in patients with neuropathic and in patients with non-neuropathic pain based on a conventional single assessment by one physician. At a optimal cut off point of ≥18 sensitivity was 83% and the specificity was 84%.

In a cohort of patients with a spinal cord injury for more than one year, pain lasting more than six months and a pain intensity of than more three on a NRS (0–10) a sensitivity was found of 68% and specificity of 83% [50].

The present study included patients with chronic pain without limits to the minimal pain intensity or other limitations. At the moment of inclusion in the study our patients had only a provisional diagnosis (LBLP, NSA pain, suspected PND pain) established in primary or secondary care without further refinement or confirmation. Then, after referral to a (non-) academic pain clinic, they were assessed as to their complaints for the first time at study inclusion. Thus this was a ‘real-life’ clinical out-patient population. Avoiding patient selection due to pre-stratification to the outcome target makes our study unique and clinically more relevant as compared to other studies on the same topic and is crucial for the validation of a screening instrument.

### Validation studies with patients not pre-stratified for NePC

In a study by Gauffin et al. [51] in patients diagnosed with fibromyalgia (*n* = 158) a cut-off score of 17 was found with a sensitivity of 79% and specificity of 53% (Gold standard: the classification by one experienced physician). This study, like ours, did not pre-stratify patients according to the pain classification either, and patients were not excluded because of a low pain level. The outcome of Gauffin’s sensitivity analyses in this fibromyalgia study was comparable to our study. Tampin et al. [34] found, based on the examination by a physical therapist, a sensitivity and specificity of the Pain*DETECT* of respectively 64 and 62% (cut-off score 18.5) in a population of patients with neck/upper limb pain (*n* = 122). In our study, the outcome for patients with neck-shoulder-arm pain was 83 and 44% respectively (cut-off score of ≥9).

### Grading system

In this study the physicians assessed patients for the presence of a NePC according to the Grading System [31]. Probable neuropathic pain and definite neuropathic pain were combined as present-NePC, and non neuropathic pain and possible neuropathic pain were combined in absent-NePC. Sensitivity and specificity of the Pain*DETECT*_-Dlv_ resulted to be 74 and 46% respectively (Cut-off score 11 out of 38, *n* = 232). Using the classification of patients’ pain based solely on the Grading System by one physician results in a lower validity than based on the physicians assessment. This might suggest that the classification of patients’ pain based on the Grading System is less accurate than the classification based on the physicians’ assessment in respect to the outcome of the Pain*DETECT*_-Dlv_. However, the grading system was assessed by the same physician who also performed the physician’s assessment so it is also possible that the physician had difficulties to classify patients pain based on the Grading System or vice versa. When using the physicians’ assessments as well as the Grading Systems of both physicians (*n* = 161), sensitivity was 78%, specificity was 53% and the cut-off score was 11: The same poor result as for the gold standard. In the papers by Vaegter et al. [33] and Tampin et al. [34] the Pain*DETECT* was also compared with the NeuPSIG Grading System. In both papers, like in ours, the outcome of the Grading System was not comparable to the outcome of the Pain*DETECT*. As stated by Finnerup et al. [52] the Pain*DETECT* (and other screening tools for the assessment of neuropathic pain) is only to alert the physician to further assess the patient who may have a NePC.

### NePC classification

The initial classification of patients’ pain in our study was based on an interview and (clinical/physical) examination by trained (pain-) physicians. There is a lack of consensus with respect to the classification of a NePC in patients with pain of different origins [53]. Moreover, a lack of standardization of assessment methods increases the number of undetected or poorly classified patients which leads to a variation in the classification accuracy (i.e. sensitivity and specificity) of screening tools caused by differences in strategy and patient population [15, 54]. Bouhassira and Attal recently stated that neuropathic pain is *“a consistent clinical entity, but it is multidimensional in terms of its clinical expression, with different sensory profiles, potentially reflecting specific pathophysiological mechanisms*” [55]. As stated by Scholz et al. [53] physical tests are more useful to identify patients with neuropathic back pain than interview questions. To reach a more unified classification system to differentiate between present-NePC and absent-NePC a standardized assessment of symptoms and signs is necessary [53]. However, these tests are not able to confirm the relation between the potential lesion or disease of the nerve and the pain directly: The classification of neuropathic pain should be based on clinical examination and the interpretation should be placed in the clinical context of patients’ pain [55].

In this study we used a mandatory standardized assessment [25] in addition to the medical history and physical examination which were performed according to the physicians’ standards. The clinical assessment and the use of the Grading System showed that in 18–22% of the patients a non-consistent assessments was present resulting in an ‘undetermined’ status. In Freynhagens paper [2] it was 5%. This difference might be due to the inclusion of patients with a less clear absent or present NePC in our study which might reflect what happens in the assessment of a NePC in usual clinical care. Moreover, this also might occur in the treatment of patients with chronic pain. Based on both the physician’s assessments, almost 75% of the patients in this study had a neuropathic pain component. This might be due to several facts. (1) Patients with LBLP or NSA pain were only included when the pain was radiating into the leg(−s) respectively the arm(−s) and were not removed from this study when they had mixed pain. Moreover, patients with radiating pain are more suspected to have a NePC. (2) There is a possibility that neuroplastic changes are interpreted as neuropathy in patients with chronic LBLP. (3) Patients were recruited in secondary and tertiary pain clinics. This might have led to a inclusion of patients who were more difficult to treat in primary care and (4) we included 108 patients with suspected peripheral nerve damage. Almost 60% of the patients after treatment for breast cancer has pain [56]. Based on the recent review by Ilhan et al. [57], in patients who reported pain following breast cancer treatment the pooled prevalence of neuropathic pain from screening questionnaires ranged from 32.6 to 58.2%. Following the NeuPSIG Grading System the prevalence ranged from 29.5 to 57.1%. Based on these numbers, patients after breast cancer can be regarded as patients suspected of neuropathic pain due to peripheral nerve damage. However, the Pain*DETECT*_-Dlv_ (compared to the gold standard and the NeuPSIG Grading System) as used in our study seems not valid for the assessment of patients with neuropathic pain based on a suspected PND in which the majority of patients was suffering of pain after treatment for breast cancer.

### Strengths and weaknesses

There are several strengths in this study. Firstly, we included a large population of patients with diagnoses who are regularly seen in daily clinical practice. Secondly, there was no pre-stratification on the target outcome, clear inclusion criteria and almost no exclusion criteria. Thirdly, we used the NeuPSIG Grading System [31, 52] as a secondary comparison. The main purpose of the Grading System is to help in the classification of the pain as neuropathic [52]. In our study, the Grading System was added to the standardized assessment form which had to be filled in by the physician. There are also some weaknesses in this study. The use of the Grading System within the clinical assessment (including bed-side examination) is a strong aspect of our study, but the outcome of the clinical examination as well as the outcome of the Grading System might be influenced by each other. However, combining the physicians’ assessment with the Grading System might have made the ‘gold standard’ even stronger but also might have led to a cross-contamination. Secondly, diagnosing NePC by assessing patients’ pain by two separate physicians in our and in other studies is considered as the ‘Gold Standard’. However, classifying patients’ pain may be done more objectively by establishing a damaged nerve and by diagnosticating in a more detailed clinical way. Moreover, the breakdown of clinical grounds for in- and exclusion could also have been assessed and captured in more detail. Thirdly, 62 physicians participated. This might have led to the inclusion of younger, less clinical experienced physicians. However, it reflects ‘real life’ practice and limits the risk of systematic bias in the classification of patients’ pain and bias based on assumptions about the existence of a NePC. Moreover, all physicians followed the standardized training as described. Fourthly, almost only patients with peripheral causes of pain were selected. This can be considered as a methodological drawback. Moreover, because we did not include patients with, by example, low back pain without irradiation to the leg who would probably be diagnosed as absent-NePC the specificity might decrease. Fifthly, there is an apparent lack of objective tests to determine whether the somatosensory fibers were affected, in particular the small fibers. This can be seen as crucial since objective data are mandatory to reach a definite neuropathic pain classification in the grading system. Lastly, in a following study we would collect data from the patients who were not able to participate in the study to prevent inclusion bias. In this study this was not possible because of ethical regulations.

## Conclusions

The Pain*DETECT*_-Dlv_ has a good internal consistency and test-retest reliability but is not an effective screening tool for the assessment of a neuropathic pain component in a population of patients with chronic pain, irrespective of the chosen comparison because of its moderate sensitivity and low specificity. However, the agreement by both the physicians and the agreement with the grading systems (performed by the physicians) were also not impressive. Moreover, the differences in the cut-off scores for the different comparisons reflects the fact that agreement in a not pre-stratified to the target outcome patient population is not easy to accomplish. Using the Pain*DETECT*_-Dlv_ (for screening purposes or as a surrogate for clinical assessment) may result in unreliably separating NePC presence from non-presence in patients with chronic pain in clinical outpatient practices and in research settings. Catch-22 situations in the validation of screening tools can be prevented by not pre-stratifying the patients on basis of the target outcome before inclusion in a validation study for screening instruments. For now, classifying patients pain still needs the clinical assessment based on history and physical examination including bed-side sensory testing by the physician and cannot be replaced by the use of the PainDETECT.

## Additional file

Additional file 1: **Table S1.** The AUC and the sensitivity / specificity at the optimal cut-off point of the PainDETECT under the condition of equal costs of misclassification to classify a NePC by the diagnosis and the grading system of the physicians for the total group and according the pain locations. (PDF 423 kb)

## Abbreviations

AUC: Area Under the Curve
BMI: Body Mass Index
CI: Confidence Interval
DRI: Disability Rating Index
EFNS: European Federation of Neurological Societies
HADS: Hospital Anxiety and Depression Scale
IASP: International Association for the Study of Pain
ICC: Intraclass Correlation
ICD-10: International Statistical Classification of Diseases and Related Health Problems 10th revision
IQR: Inter Quartile Range
K: Cohen’s kappa
LBLP: Low Back with Leg Pain
NePC: Neuropathic Pain Component
NeuPSIG: Neuropathic Pain Special Interest Group
NND: Number Needed to Diagnose
NRS: Numeric Rating Scale
NSA pain: Neck-Shoulder-Arm pain
NTR: National Trial register
PA: Percentage of Agreement
Pain*DETECT*_-Dlv_: Pain*DETECT* Dutch language version
PAS: Pain Attribution Scale
PGIC: Patient Global Impression of Change
P*i*: Prevalence index
PND: Peripheral nerve damage pain
RAND-36: RAND 36-item health Survey
ROC: Receiver Operating Characteristic
SD: Standard Deviation
SPSS: Statistical package for the Social Sciences
VAS: Visual Analogue Scale
WHO: World Health Organization
