# Supplementary material for: Avoiding Catch-22: validating the PainDETECT in a in a population of patients with chronic pain
Source: BMC Neurol. 2018 Jun 29;18:91. doi: 10.1186/s12883-018-1094-4 (PMC6026336; doi:10.1186/s12883-018-1094-4)
Supplement: Supplementary file 1 — Table S1. The AUC and the sensitivity / specificity at the optimal cut-off point of the PainDETECT under the condition of equal costs of misclassification to classify a NePC by the diagnosis and the grading system of the physicians for the total group and according the pain locations. (PDF 423 kb) [file 12883_2018_1094_MOESM1_ESM.pdf]

Additional file 1: Table S1: The AUC and the sensitivity / specificity at the optimal cut-off point of the PainDETECT under the condition of equal costs of misclassification to classify a NePC by the diagnosis and the grading system of the physicians for the total group and according the pain locations

	NePC	Absent NePC	AUC	(95%CI)	Youden index	Cut-off	T+	F+	F-	T-	Sens %	95% CI	Spec %	95% CI	NND	PPV %	NPV %	PLR	95% CI	NLR	95% CI	DOR	95% CI	P[Z+]	P[Z-]	FPR %	FNR %
**Diagnosis**																											
**A**	**208**	**83**	**69.8**	**(0.631-0.766)**	**0.318**	**9**	**179**	**45**	**29**	**38**	**86.06**	**(0.807 -0.901)**	**45.78**	**(0.355-0.565)**	**3.14**	**79.91**	**56.72**	**1.59**	**(1.293-1.949)**	**0.30**	**(0.202-0.459)**	**5.21**	**(2.908-9.341)**	**71.48**	**28.52**	**54.22**	**13.94**
LBLP (n)	95	37	71.7	(0.621-0.813)	0.381	12	67	12	28	25	70.53	(0.607-0.788)	67.57	(0.515-0.804)	2.63	84.81	47.17	2.17	(1.342-3.524)	0.44	(0.297-0.64)	4.99	(2.201-11.29)	71.97	28.03	32.43	29.47
NSA PAIN (n)	28	23	62.3	(0.463-0.783)	0.335	9	24	12	4	11	85.71	(0.685-0.943)	47.83	(0.292-0.670)	2.98	66.67	73.33	1.64	(1.08-2.499)	0.30	(0.11-0.814)	5.5	(1.443-20.959)	54.90	45.10	52.17	14.29
sPND (n)	85	23	72.3	(0.610-0.836)	0.344	15	83	19	2	4	97.65	(0.918-0.994)	17.39	(0.07-0.371)	6.65	81.37	66.67	1.18	(0.977-1.43)	0.14	(0.026-0.693)	8.74	(1.49-51.244)	78.70	21.30	82.61	2.35
KPND (n)	21	1	90.5	(0.779-1.000)	0.905	7	19	0	2	1	90.48	(0.711-0.974)	100	(0.207-1)	1.11	100	33.33	---	---	0.10	(0.025-0.356)	---	---	95.45	4.55	0.00	9.52
Breast (n)	64	22	72.4	(0.601-0.848)	0.334	15	33	4	31	18	51.56	(0.396-0.634)	81.82	(0.615-0.927)	3.00	89.19	36.73	2.84	(1.133-7.1)	0.59	(0.43-0.816)	4.79	(1.459-15.733)	74.42	25.58	18.18	48.44
**B**	**195**	**96**	**67.2**	**(0.609-0.735)**	**0.275**	**11**	**147**	**46**	**48**	**50**	**75.38**	(0.689-0.809)	**52.08**	**(0.422-0.618)**	**3.64**	**76.17**	**51.02**	**1.57**	**(1.258-1.967)**	**0.47**	**(0.346-0.645)**	**3.33**	**(1.987-5.578)**	**67.01**	**32.99**	**47.92**	**24.62**
LBLP (n)	84	48	70.9	(0.619-0.799)	0.423	11	67	18	17	30	79.76	(0.7-0.87)	62.5	(0.484-0.748)	2.37	78.82	63.83	2.13	(1.453-3.113)	0.32	(0.201-0.522)	6.57	(2.98-14.479)	63.64	36.36	37.50	20.24
NSA PAIN (n)	28	23	58.7	(0.430-0.744)	0.219	17	11	4	17	19	39.29	(0.236-0.5)76	82.61	(0.629-0.930)	4.57	73.33	52.78	2.26	(0.829-6.157)	0.73	(0.517-1.045)	3.07	(0.823-11.485)	54.90	45.10	17.39	60.71
sPND (n)	83	25	66.3	(0.558-0.768)	0.326	16	37	3	46	22	44.58	(0.344-0.55)3	88.00	(0. 7-0.958)	3.07	92.50	32.35	3.71	(1.251-11.03)	0.63	(0.495-0.802)	5.9	(1.637-21.248)	76.85	23.15	12.00	55.42
KPND (n)	21	1	69.0	(0.491-0.890)	0.667	11	14	0	7	1	66.67	(0.454-0.828)	100	(0. 207-1)	1.50	100	12.50	---	---	0.33	(0.182-0.61)	---	---	95.45	4.55	0.00	33.33
Breast (n)	62	24	66.8	(0.552-0.784)	0.310	16	27	3	35	21	43.55	(0.319-0.559)	87.50	(0.69-0.957)	3.22	90.00	37.50	3.48	(1.165-10.422)	0.65	(0.495-0.842)	5.4	(1.457-20.008)	72.09	27.91	12.50	56.45
**A=B**	**170**	**58**	**72.1**	**(0.646-0.796)**	**0.352**	**11**	**136**	**26**	**34**	**32**	**80.00**	(0.734-0.853)	**55.17**	**(0.425-0.673)**	**2.84**	**83.95**	**48.48**	**1.78**	(1.328-2.398)	**0.36**	(0.248-0.53)	**4.92**	**(2.597-9.332)**	**74.56**	**25.44**	**44.83**	**20.00**
LBLP (n)	75	28	75.4	(0.650-0.858)	0.483	11	63	10	12	18	84.00	(0.741-0.906)	64.29	(0.458-0.793)	2.07	86.30	60.00	2.35	(1.417-3.904)	0.25	(0.138-0.448)	9.45	(3.514-25.414)	72.82	27.18	35.71	16.00
NSA PAIN (n)	23	18	62.9	(0.455-0.804)	0.271	9	19	10	4	8	82.61	(0.629-0.930)	44.44	(0.246-0.663)	3.70	65.52	66.67	1.49	(0.945-2.341)	0.39	(0.14-1.096)	3.8	(0.915-15.779)	56.10	43.90	55.56	17.39
sPND (n)	72	12	75.5	(0.632-0.878)	0.472	15	40	1	32	11	55.56	(0.441-0.665)	91.67	(0.646-0.985)	2.12	97.56	25.58	6.67	(1.009-44.035)	0.48	(0.356-0.661)	13.75	(1.685-112.198)	85.71	14.29	8.33	44.44
KPND (n)	20	0	---	---	---	---	---	---	---	---	---	---	---	---	---	---	---	---	---	---	---	---	---		---	---	---
Breast (n)	52	12	77.1	(0.640-0.902)	0.472	15	29	1	23	11	55.77	(0.423-0.684)	91.67	(0.646-0.985)	2.11	96.67	32.35	6.69	(1.009-44.391)	0.48	(0.483-0.0684)	13.87	(1.666-115.434)	81.25	18.75	8.33	44.23
**Grading**																											
**A**	**172**	**114**	**58.9**	**(0.520-0.658)**	**0.171**	**11**	**126**	**64**	**46**	**50**	**73.26**	**(0.662-0.793)**	**43.86**	**(0.351-0.53)**	**5.84**	**66.32**	**52.08**	**1.30**	**(1.084-1.571)**	**0.61**	**(0.441-0.842)**	**2.14**	**(1.297-3.531)**	**60.14**	**39.86**	**56.14**	**26.74**
LBLP (n)	62	69	60.6	(0.509-0.704)	0.248	12	45	33	17	36	72.58	(0.604-0.821)	52.17	(0.406-0.635)	4.04	57.69	67.92	1.52	(1.135-2.028)	0.53	(0.331-0.836)	2.89	(1.39-5.999)	47.33	52.67	47.83	27.42
NSA PAIN (n)	17	**30**	50.3	(0.329-0.677)	0.127	18	5	5	12	25	29.41	(0.133-0.531)	83.33	(0.664-0.927)	7.85	50.00	67.57	1.76	(0.595-5.235)	0.85	(0.599-1.197)	2.08	(0.505-8.601)	36.17	63.83	16.67	70.59
SPND (n)	93	**15**	63.0	(0.467-0.793)	0.335	13	56	4	37	11	60.22	(0.501-0.696)	73.33	(0.481-0.891)	2.98	93.33	22.92	2.26	(0.96-5.311)	0.54	(0.366-0.805)	4.16	(1.232-14.061)	86.11	13.89	26.67	39.78
KPND (n)	16	**6**	51.6	(0.237-0.795)	0.292	13	10	2	6	4	62.50	(0.386-0.815)	66.67	(0.3-0.903)	3.43	83.33	40.00	1.88	(0.568-6.185)	0.56	(0.241-1.314)	3.33	(1.462-24.052)	72.73	27.27	33.33	37.50
Breast (n)	77	**9**	69.7	(0.508-0.886)	0.375	13	46	2	31	7	59.74	(0.486-0.7)	77.78	(0.453-0.937)	2..67	95.83	18.42	2.69	(0.781-9.252)	0.52	(0.332-0.806)	5.19	(1.011-26.67)	89.53	10.47	22.22	40.26
**B**	**158**	**130**	**58.6**	**(0.520-0.652)**	**0.168**	**14**	**91**	**53**	**67**	**77**	**57.59**	**(0.498-0.650)**	**59.23**	**(0.506-0.673)**	**5.94**	**63.19**	**53.47**	**1.41**	**(1.104-1.808)**	**0.72**	**(0.568-0.902)**	**1.97**	**(1.232-3.161)**	**54.86**	**45.14**	**40.77**	**42.41**
LBLP (n)	56	75	62.3	(0.524-0.722)	0.216	14	36	32	20	43	64.29	(0.512-0.755)	57.33	(0.461-0.679)	4.63	52.94	68.25	1.51	(1.086-2.09)	0.62	(0.417-0.931)	2.42	(1.186-4.934)	42.75	57.25	42.67	35.71
NSA PAIN (n)	19	32	48.7	(0.311-0.663)	0.191	19	6	4	13	28	31.58	(0.154-0.54)	87.50	(0.719-0.95)	5.24	60.00	68.29	2.53	(0.816-7.826)	0.78	(0.561-1.09)	3.23	(0.776-13.446)	37.25	62.75	12.50	68.42
sPND (n)	83	23	59.1	(0.458-0.724)	0.279	13	9	16	10	16	47.37	(0.273-0.683)	50.00	(0.336-0.664)	-38	36.00	61.54	0.95	(0.527-1.704)	1.05	(0.608-1.824)	0.9	(0.289-2.804)	37.25	62.75	50.00	52.63
KPND (n)	15	6	49.4	(0.213-0.776)	0.267	14	9	2	6	4	60.00	(0.358-0.802)	66.67	(0.3-0.903)	3.75	81.82	40.00	1.80	(0.54-6.004)	0.60	(0.259-1.389)	3	(0.411-21.881)	71.43	28.57	33.33	40.00
Breast (n)	68	17	62.6	(0.476-0.776)	0.338	13	43	5	25	12	63.24	(0.514-0.737)	70.59	(0.469-0.867)	2.96	89.58	32.43	2.15	(1.007-4.59)	0.52	(0.336-0.807)	4.13	(1.302-13.088)	80.00	20.00	29.41	36.76
**A=B**	**139**	**93**	**61.3**	**(0.539-0.687)**	**0.203**	**11**	**103**	**50**	**36**	**43**	**74.10**	**(0.662-0.807)**	**46.24**	**(0.365-0.563)**	**4.92**	**67.32**	**54.43**	**1.38**	**(1.114-1.705)**	**0.56**	**(0.392-0.8)**	**2.46**	**(1.41-4.294)**	**59.91**	**40.09**	**53.76**	**25.90**
LBLP (n)	48	60	63.1	(0.524-0.739)	0.258	12	34	27	14	33	70.83	(0.568-0.818)	55.00	(0.425-0.669)	3.87	55.74	70.21	1.57	(1.128-2.197)	0.53	(0.323-0.87)1	2.97	(1.329-6.631)	44.44	55.56	45.00	29.17
NSA PAIN (n)	13	24	48.9	(0.284-0.694)	0.183	18	4	3	9	21	30.77	(0.127-0.576)	87.50	(0.69-0.957)	5.47	57.14	70.00	2.46	(0.647-9.365)	0.79	(0.534-1.172)	3.11	(0.575-16.833)	35.14	64.86	12.50	69.23
sPND (n)	78	9	66.0	(0.453-0.867)	0.419	13	50	2	28	7	64.10	(0.53-0.739)	77.78	(0.453-0.937)	2.39	96.15	20.00	2.88	(0.84-9.903)	0.46	(0.292-0.73)	6.25	(1.215-32.16)	89.66	10.34	22.22	35.90
KPND (n)	14	4	50.9	(0.159-0.859)	0.321	14	8	1	6	3	57.14	(0.326-0.786)	75.00	(0.301-0.954)	3.11	88.89	33.33	2.29	(0.394-13.245)	0.57	(0.25-1.308)	4	(0.329-48.656)	77.78	22.22	25.00	42.86
Breast (n)	64	5	71.3	(0.486-0.939)	0.456	13	41	1	22	4	65.08	(0.528-0.757)	80.00	(0.376-0.964)	2.22	97.62	15.38	3.25	(0.559-18.958)	0.44	(0.251-0.759)	7.46	(0.784-70.847)	92.56	7.35	20.00	34.92
**Diagnosis A = Grading A**	**155**	**63**	**69.1**	**(0.610-0.771)**	**0.317**	**11**	**118**	**28**	**37**	**35**	**76.13**	**(0.688-0.822)**	**55.56**	**(0.433-0.672)**	**3.16**	**80.82**	**48.61**	**1.71**	**(1.282-2.289)**	**0.43**	**(0.301-0.614)**	**3.99**	**(2.146-7.404)**	**71.10**	**28.90**	**44.44**	**23.87**
LBLP (n)	60	35	71.4	(0.607-0.821)	0.407	12	45	12	15	23	75.00	(0.628-0.842)	65.71	(0.492-0.792)	2.46	78.95	60.53	2.19	(1.352-3.54)	0.38	(0.231-0.627)	5.75	(2.314-14.289)	63.16	36.84	34.29	25.00
NSA PAIN (n)	15	18	56.1	(0.360-0.763)	0.244	9	12	10	3	8	80.00	(0.548-0.93)	44.44	(0.246-0.663)	4.09	54.55	72.73	1.44	(0.887-2.338)	0.45	(0.144-1.402)	3.2	(0.666-15.381)	45.45	54.55	55.56	20.00
sPND (n)	80	10	74.2	(0.578-0.907)	0.438	13	51	2	29	8	63.75	(0.528-0.734)	80.00	(0.49-0.943)	2.29	96.23	21.62	3.19	(0.913-11.132)	0.45	(0.296-0.693)	7.03	(1.399-35.374)	88.89	11.11	20.00	36.25
KPND (n)	16	1	87.5	(0.713-1.000)	0.875	7	14	0	2	1	87.50	(0.64-0.965)	100	(0.207-1)	1.14	100	33.33	---	---	0.13	(0.034-0.457)	---	---	94.12	5.88	0.00	12.50
Breast (n)	64	9	72.8	(0.543-0.913)	0.418	13	41	2	23	7	64.06	(0.518-0.747)	77.78	(0.453-0.937)	2.39	95.35	23.33	2.88	(0.838-9.922)	0.46	(0.286-0.746)	6.24	(1.195-32.563)	87.67	12.33	22.22	35.94
**Diagnosis B = Grading B**	**139**	**77**	**67.1**	**(0.598-0.743)**	**0.282**	**11**	**106**	**37**	**33**	**40**	**76.26**	**(0.685-0.826)**	**51.95**	**(0.41-0.628)**	**3.55**	**74.13**	**54.79**	**1.59**	**(1.236-2.038)**	**0.46**	**(0.317-0.66)**	**3.47**	**(1.918-6.288)**	**64.35**	**35.65**	**48.05**	**23.74**
LBLP (n)	50	42	72.3	(0.619-0.827)	0.411	11	42	18	8	24	84.00	(0.715-0.917)	57.14	(0.422-0.709)	2.43	70.00	75.00	1.96	(1.354-2.836)	0.28	(0.141-0.557)	7	(2.648-18.506)	54.35	45.65	42.86	16.00
NSA PAIN (n)	16	20	55.2	(0.354-0.749)	0.275	19	6	2	10	18	37.50	(0.185-0.614)	90.00	(0.699-0.972)	3.64	75.00	64.29	3.75	(0.872-16.132)	0.69	(0.462-1.043)	5.4	(0.913-31.933)	44.44	55.56	10.00	62.50
sPND (n)	73	15	69.2	(0.572-0.812)	0.430	13	46	3	27	12	63.01	(0.516-0.732)	80.00	(0.548-0.93)	2.32	93.88	30.77	3.15	(1.128-8.8)	0.46	(0.312-0.689)	6.82	(1.764-26.326)	82.95	17.05	20.00	36.99
KPND (n)	15	1	63.3	(0.387-0.880)	0.600	12	9	0	6	1	60.00	(0.358-0.802)	100	(0.207-1)	1.67	100	14.29	---	---	0.40	(0.215-0.743)	---	---	93.75	6.25	0.00	40.00
Breast (n)	58	14	70.1	(0.568-0.835)	0.424	13	37	3	21	11	63.79	(0.509-0.75)	78.57	(0.524-0.924)	2.36	92.50	34.38	2.98	(1.072-8.269)	0.46	(0.297-0.714)	6.46	(1.618-25.791)	80.56	19.44	21.43	36.21
**Diagnosis A = Grading A = Diagnosis B = Grading B**	**118**	**43**	**67.9**	**(0.586-0.772)**	**0.315**	**11**	**92**	**20**	**26**	**23**	**77.97**	**(0.697-0.845)**	**53.49**	**(0.389-0.675)**	**3.18**	**82.14**	**46.94**	**1.68**	**(1.2-2.342)**	**0.41**	**(0.266-0.639)**	**4.07**	**(1.94-8.535)**	**73.29**	**26.71**	**46.51**	**22.03**
LBLP (n)	43	26	73.2	(0.610-0.853)	0.453	11	36	10	7	16	83.72	(0.700-0.919)	61.54	(0.425-0.776)	2.21	78.26	69.57	2.18	(1.315-3.602)	0.26	(0.126-0.556)	8.23	(2.655-25.505)	62.32	37.68	38.46	16.28
NSA PAIN (n)	12	12	48.3	(0.244-0.721)	0.167	18	4	2	8	10	33.33	(0.138-0.609)	83.33	(0.552-0.953)	6.00	66.67	55.56	2.00	(0.448-8.936)	0.80	(0.498-1.284)	2.5	(0.361-17.315)	50.00	50.00	16.67	66.67
sPND (n)	63	5	72.5	(0.521-0.929)	0.467	13	42	1	21	4	66.67	(0.544-0.771)	80.00	(0.376-0.964)	2.14	97.67	16.00	3.33	(0.572-19.408)	0.42	(0.238-0.73)	8	(0.841-76.133)	92.65	7.35	20.00	33.33
KPND (n)	14	0	---	---	---	---	---	---	---	---	---	---	---	---	---	---	---	---	---	---	---	---	---	---	---	---	---
Breast (n)	49	5	74.7	(0.535-0.959)	0.494	13	34	1	15	4	69.39	(0.558-0.805)	80.00	(0.376-0.964)	2.02	97.14	21.05	3.47	(0.595-20.224)	0.38	(0.208-0.703)	9.07	(0.933-88.108)	90.74	9.26	20.00	30.61

PD-Q: PainDETECT questionnaire; NEPC: Neuropathic pain component existing; Absent-NePC: Neuropathic pain component not existing; AUC: Area under curve; 95%CI: 95% confidence interval; T+: True positive; F+: False positive; F-: False negative; T-: True negative; Sens.: Sensitivity; Spec.: Specificity; NND: Number needed to diagnose; +PPV: Positive predictive value; -NPV: Negative predictive value; PLR: Positive likelihood ratio; NLR: Negative likelihood ratio; DOR: Diagnostic odds ratio; P[Z+]:a-priori chance for the existence of a NePC; P[Z-]:a-priori chance for no existence of a NePC; FPR: False positive ratio; FNR: False negative ratio; A: Physician A; B: Physician B; LBLP: Patients suffering from low back pain with leg pain; NSA PAIN: Patients suffering from neck shoulder pain with arm pain; sPND: Patients suffering from a suspected periferal nerve damage; KPND: Patients suffering from a known peripheral nerve damage; Breast: patients suffering of pain after breast cancer treatment.
